# Supplementary material for: Carbon Nanodots as Electron Transport Materials in Organic Light Emitting Diodes and Solar Cells
Source: Nanomaterials (Basel). 2022 Dec 30;13(1):169. doi: 10.3390/nano13010169 (PMC9823923; doi:10.3390/nano13010169)
Supplement: Supplementary file 1 [file nanomaterials-13-00169-s001.zip › nanomaterials-2111461-supplementary.pdf]

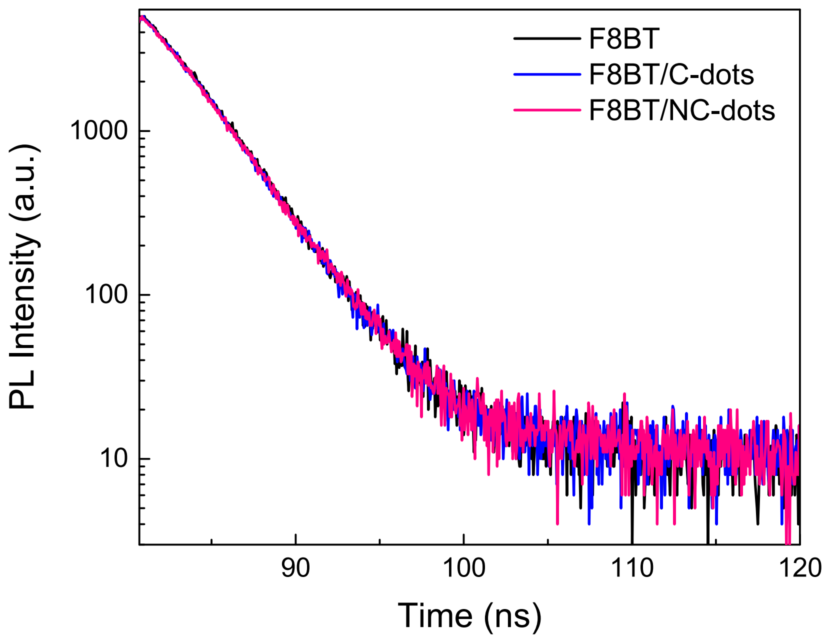

Figure S1 Time-resolved photoluminescence (TRPL) spectra of F8BT, F8BT/C-dots, and F8BT/NC-dots.

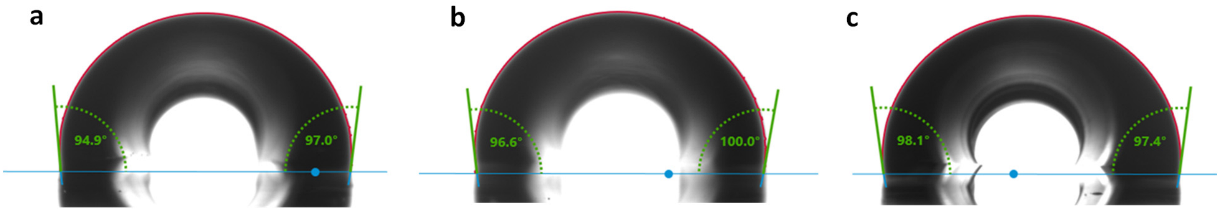

Figure S2 Water contact angle measurements taken in (a) F8BT, (b) F8BT/C-dots and (c) F8BT/NC-dots.

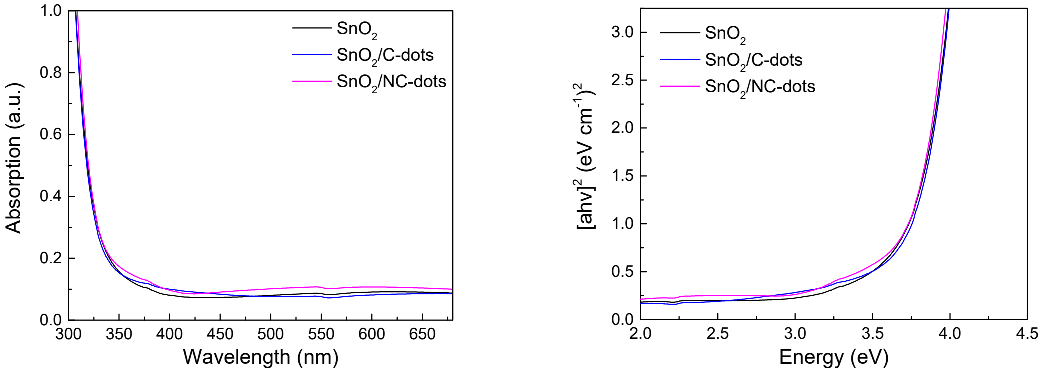

(a) **(b)**

Figure S3 (a) Absorption spectra and (b) tauc plots of pristine and nanodots-coated SnO_2.
